# Privacy-preserving augmentation of structured telehealth activity data in diabetes patients using natural language processing

**DOI:** 10.3389/fdgth.2026.1720149

**Published:** 2026-05-07

**Authors:** Fabian Wiesmüller, Karl Kreiner, Valerie Reinisch, Florian Hoffmann, Günter Schreier, Dieter Hayn

**Affiliations:** 1 Center for Health & Bioresources, AIT Austrian Institute of Technology GmbH, Graz, Austria; 2 Ludwig Boltzmann Institute for Digital Health and Prevention, Ludwig Boltzmann Gesellschaft, Salzburg, Austria; 3 Institute of Neural Engineering, Graz University of Technology, Graz, Austria; 4 Versicherungsanstalt für öffentlich Bedienstete, Eisenbahnen und Bergbau – BVAEB, Vienna, Austria

**Keywords:** free text, health notes, large language models, mHealth, natural language processing, physical activity, telehealth

## Abstract

**Introduction:**

Diabetes management increasingly relies on telehealth platforms in which patients generate structured and unstructured data. This unstructured data, in the form of free-text notes often contain additional information beyond the structured data. Extracting this information can enhance patient profiles and optimize treatment. In particular, the extraction of physical activity information from these notes is considered important. This study evaluates rule-based/regex algorithms and a locally deployed Mistral LLM for physical activity information extraction and data augmentation, with their performances benchmarked against a state-of-the-art GPT-4.1.

**Methods:**

Data from 943 patients collected over 12 years in the DiabMemory system, supplemented by 100 synthetic notes, were analyzed. Patients’ privacy was preserved by applying a free text pseudonymization algorithm to all notes and by using locally deployed approaches, thereby avoiding third-party cloud services. Three tasks were conducted: (1) extraction of physical activity (PA) data from free-text notes using regex and a locally deployed Mistral LLM, (2) integration of extracted data with structured activity records using a rule-based approach and the local Mistral LLM, and (3) benchmarking local approaches against GPT-4.1 based on the synthetic notes.

**Results:**

Both local methods achieved strong performance in task 1, with minimum F1-scores of 0.84. In task 2, rule-based augmentation (F1 = 0.73) surpassed the Mistral LLM (F1 = 0.37). Task 3 showed GPT-4.1 outperforming the local LLM but not consistently surpassing regex. The rule-based algorithms also required substantially less computation time than either LLM.

**Discussion:**

The regex algorithm achieved superior accuracy and efficiency but required extensive dataset-specific development, while prompt engineering for the LLM required less knowledge and the development time for regex exceeded that of LLM prompt engineering. Findings of this work generally align with prior studies but are limited by the rather small test set and use of synthetic data.

**Conclusions:**

Local NLP approaches can enhance structured PA data in diabetes telehealth. Rule-based algorithms remain a strong option where computational resources are limited, though future work should validate these findings on larger and more diverse datasets.

## 1 Introduction

Diabetes is one of the most prevalent chronic diseases with an estimated 600,000 affected people in Austria and approximately 828 million people globally. The number of undiagnosed individuals is believed to be even higher. Between 1990 and 2022 the number of estimated people living with Diabetes has more than quadrupled with a rapid increase in low to middle income countries (1–3).

Since these numbers put a lot of financial and administrative strain on healthcare systems (4), cost effective and easy to implement treatment methods become increasingly important and necessary. One such treatment is the increase in physical activity (PA), which has shown a high effectiveness in managing chronic diseases, with healthcare professionals (HCP) going as far as to prescribe PA as a treatment option (5, 6). The 2025 Standards of Care in Diabetes by the American Diabetes Association recommend lifestyle changes which include nutrition, physical activity, and behavioral therapy for both, as a treatment for type 2 diabetes patients and as a tool to prevent or delay the onset of diabetes. The recommended PA levels are in accordance with the WHO recommendation for physical activity (6–8).

Another increasingly important tool in Diabetes management is telehealth, which is defined as the delivery of healthcare or rehabilitation services to patients remotely using digital technologies (9). Over the last years telehealth has proven to be effective in the field of cardiovascular diseases and diabetes. Therefore, the American Diabetes Association recommends the usage of telehealth to provide enhanced timely access to diabetes care, educational content, self-management services and for an optimized glycemic management (10–13). It is, however, emphasized that telehealth tools should only be used in combination with in-person visits with a HCP (14). A diabetes disease management program has been implemented in Austria in 2010 by the Versicherungsanstalt für öffentlich Bedienstete, Eisenbahnen und Bergbau (BVAEB) in collaboration with the AIT Austrian Institute of Technology under the name DiabMemory. Within DiabMemory, patients upload data like blood sugar values, insulin administration and their daily food intake in a structured way (15). To further foster lifestyle changes via DiabMemory, patients can also manually record PA data in using type - intensity - duration triples. Therefore, a set list of activity types to choose from is provided. Intensity is recorded by a predefined scale of low, medium and high. Currently there is no way to report objective PA intensity measures like the heart rate during exercise in a structured way in DiabMemory. HCP routinely review these measurements and provide feedback to patients through free text notes. Likewise, patients can send free text notes as messages to the HCP, either to respond to their feedback messages or to add additional arbitrary information (16).

Previous work already made efforts towards analyzing and structuring these free text notes. Whilst the emphasis of this analysis was on parameters like state of health, nutrition and medication, a large potential for PA related information has been identified (17). Since the choices for activity types were limited to 7 common exercises (cycling, gymnastics, hiking, power walking, swimming, running, tennis) and a category others and the intensity level was also very limited with only 3 levels (low, medium and high), DiabMemory patients often added additional information like the specification of the type *others*, and other exercise relevant parameters like the heart rate, covered distances and even the number of steps taken in a note.

Analyzing these notes yields great potential for further information about DiabMemory patients, which might be useful for optimizing their diabetes treatment. The advancements in large language model (LLM) technology led to a boom and a shift in natural language processing (NLP) in all fields, including healthcare, and ever often LLMs are used for analyzing healthcare related notes (18–21). While the number of pretrained open source LLMs is constantly increasing, the computational power needed to deploy very large, state-of-the-art models and run them locally is substantial. Contrary to LLMs, rule-based approaches like regular expressions (regex) can be run locally with very limited computational power. Regex are based on pattern matching, which has the downside, that these patterns must be developed in a time-consuming process which requires in depth knowledge of the data which is analyzed. A comparison between rule-based approaches and local LLMs in a healthcare setting is sparse but previous work has focused on the extraction of information from clinical notes. Kaster et al. demonstrated that phenotype extraction from clinical notes on neurofibromatosis was more effective with a rule-based approach than with the tested LLM methods, although the rule-based system showed slightly lower generalizability (22). Similarly, Patra et al. compared rule-based NLP with an LLM approach for information extraction from psychiatry notes and found that the rule-based method performed better (23). In contrast, Sivarajkumar et al. reported that when comparing rule-based, machine learning, and LLM-based approaches, no single method consistently outperformed the others across all evaluated categories (24).

The aim of this work is to compare different privacy-preserving physical activity augmentation algorithms applied on free text notes. In particular, a regex-based algorithm was compared to a locally deployed LLM to extract PA related information from diabetes telehealth free text notes. The extracted data is then used to augment and complete structured PA entries. The information extraction performance was also benchmarked against a state-of-the-art GPT model using a set of synthetically generated notes.

## 2 Methods

### 2.1 Dataset

For this work data from the DiabMemory diabetes telemedicine system collected between April 2010 and February 2022 was used. The structured data export included PA data and the patient characteristics (e.g., age). Additionally, all free text notes written by patients were exported. Structured PA data consisted of the following three non-mandatory parameters:

- Duration: the duration of the activities in minutes, logged by the patient
- Intensity: the self-reported intensity of the activities on three levels (low, medium, high)
- Type: the type of activity performed (cycling, gymnastics, hiking, power walking, swimming, running, tennis, others)

Overall, 286,451 structured activity entries from 943 patients with a mean of 303.77 (±748.60) and a median of 53 (IQR: 7-247) entries per patient were recorded during the export period. Out of those, 186,867 (65.24%) were complete with valid data for all three parameters. In addition to the structured entries, 84,037 notes were uploaded by 909 patients with a mean of 92.45 (±389.81) and a median of 12 (IQR: 3–50) notes per patient. Even though this work did not share any real-world data with third parties, a locally executed pseudonymization algorithm was applied to the data beforehand, which masked personal data, to ensure high privacy standards (25).

To get a better understanding of the performance of the local methods compared to state-of-the­-art LLMs, a set of 100 synthetic notes was generated, which has been annotated by two scientists. The notes were generated with the local LLM using a sample of the actual notes as a reference, to ensure that the synthetic data represented the style and content of the actual notes.

### 2.2 Tasks for the algorithms

We developed and compared different algorithms to be applied on free text notes, which were designed to resolve two types of tasks: information retrieval and classification, and, subsequently, augmentation of existing data.

#### 2.2.1 Task 1 – information retrieval and classification

This task focused on extracting structured information from the free text notes. Following three subtasks were addressed:

- Task 1.1 – Activity classification: A classification of each note as “activity related” or “not activity related”.
- Task 1.2 – Primary parameters: The actual extraction of the three parameters which were part of the structured PA entries (type – intensity – duration).
- Task 1.3 – Secondary parameters: The extraction of additional PA relevant information which could not be uploaded in a structured way. These parameters include distance covered in kilometers, elevation changes in meters, exercise workload in watt, energy expenditure in calories, heart rate during exercise and steps taken on a given day.

**Figure 1:**
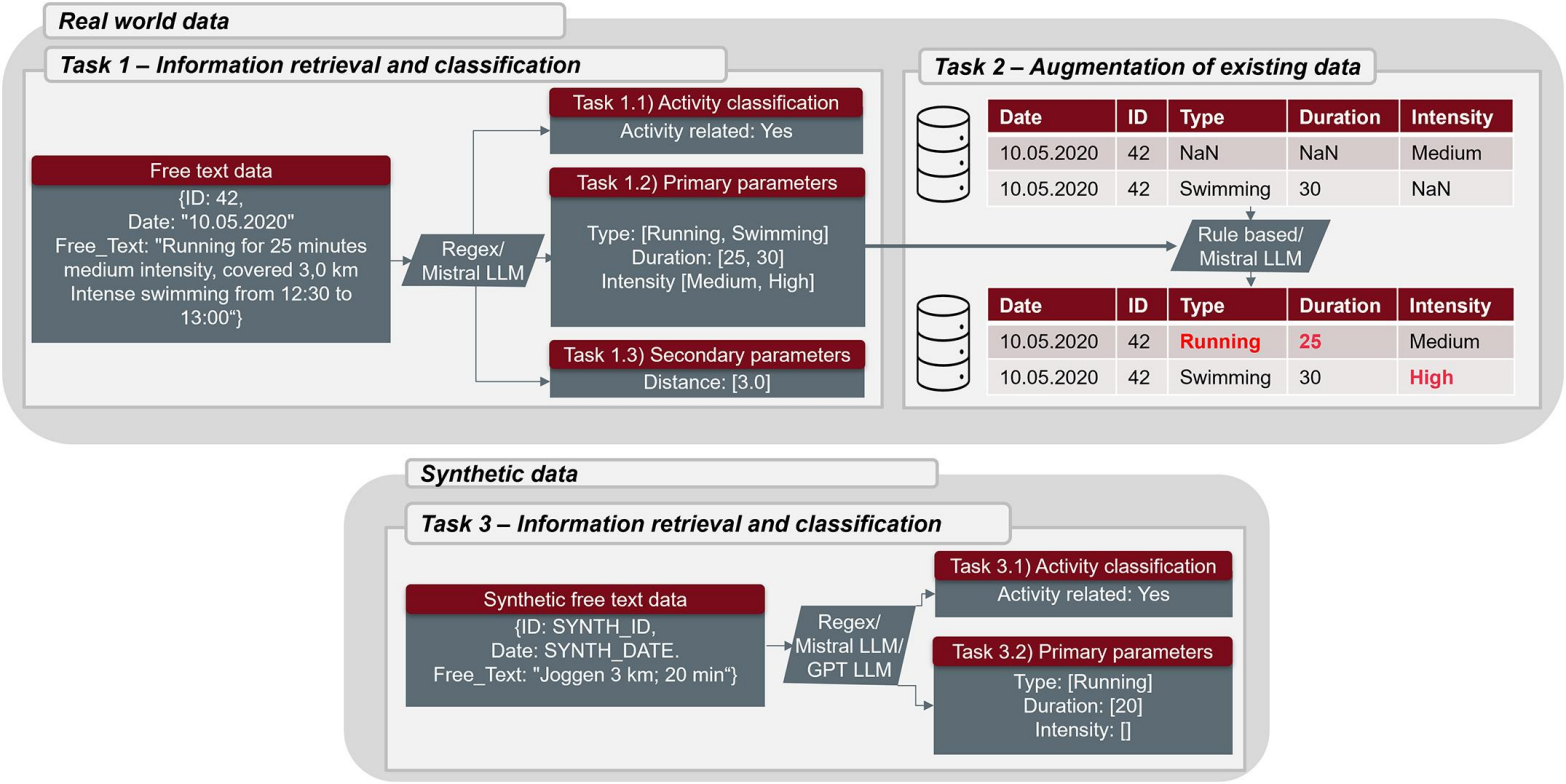
Example workflow of the tasks performed by the regular expression (regex)/rule-based algorithms and the mistral (local) and GPT (cloud-based) large language models (LLM).

The information retrieval task is illustrated with an example in Figure 1.

#### 2.2.2 Task 2 – augmentation of existing data

After the information retrieval, the algorithms should augment the structured PA data using the information gained from task 1. This augmentation had three possible scenarios:

- Missing entry: A PA was only reported in the note without any structured entry.
- Incorrect parameters: One (or more) parameter of the structured entry was further specified or corrected through the note (e.g., skiing instead of *others* or 30 minutes instead of 40 minutes).
- Incorrect entry: A structured PA was wrongfully submitted and should be removed entirely.

The data augmentation task was only applied to real-world data and is illustrated with an example in Figure 1.

#### 2.2.3 Taks 3 - information retrieval and classification with synthetic data

Like task 1, task 3 was split in subtasks, where subtask 3.1 concerned activity classification, and subtask 3.2 focused on the extraction of primary parameters. However, instead of real-world data, we used synthetic data and added the GPT model as a third extraction algorithm. Synthetic data were introduced because using real patient data with a third-party, cloud-based LLM could not be justified due to ethical and legal concerns. This task is illustrated at the bottom of Figure 1. Secondary parameters were not analyzed using the synthetic dataset based on the small size of the test set and therefore the lack of positive cases.

### 2.3 Regex-based algorithm

The regex-based algorithm was designed in Matlab^1^ and applied various regex patterns to the free text notes to retrieve the primary parameters. We manually developed 54 regex patterns based on prior experience with telehealth notes to cover relevant activity types in the free text notes. Additionally, one *negative* pattern was developed to exclude typical non-activity-related content, even if it matched one of the 54 patterns. The 54 regex patterns were then grouped into 34 more general activity classes, which was necessary for comparison with the gold standard. For example the regex pattern number 20 in the Supplementary appendix (\w*(?<!aqua|wasser|ski|schi)gymnasti(k|c)\w*|boden\s?gym\w*|(\s|^)gymn(\.|\s|$)|gymn(\.|\;|\d)) as well as regex 42 in the Supplementary appendix (aufwär\w*|aufwaerm\w*|dehnen) and *cool(\-|\s)?down* were all grouped together under the category gymnastics. During the annotation process these 34 categories proved to be sufficient for covering all activity types.

For extraction of PA duration, two slightly different, complex patterns were constructed: One pattern for notes, where an activity type as described above had previously been found, and one pattern for notes without activity types. Since the first pattern was only applied on the pre-selected, activity-related notes, it was designed more inclusively, focusing primarily on high sensitivity in duration detection. The second pattern, however, was less inclusive and required actual time indicators (e.g., minutes) in combinations with numbers and PA information, to minimize false positive classifications.

There was only a small number of intensity instances in the notes. Therefore, only four simple patterns for intensity level extraction were designed.

Most secondary parameters were characterized by clear indicators, such as numeric values followed by unit indicators (e.g., “km” for distances or “hm” for elevation changes/“Höhenmeter” in German). Therefore, only one pattern per parameter was developed.

Classification into activity related or non-activity-related notes was based on the extraction of the previous three parameters. Whenever one of the primary parameters was present in the note, it was flagged as an activity related note.

The rule-based algorithm for task 2 compared information extracted from clinical notes with existing structured data to determine whether a new entry should be created or an existing one updated. Entry deletion was handled separately and triggered by predefined regular expression patterns. To provide sufficient context, the algorithm processed all notes and all structured PA entries for a single patient on the same day as a single input, rather than handling each note or structured entry separately.

### 2.4 Augmentation of existing data

The rule-based augmentation algorithm was also implemented in Matlab^2^ and merged data from two sources: free-text notes and structured entries (see Section 2.2.2). While the initial idea was to include all 3 primary parameters for the augmentation, due to low number of notes with an intensity, for the time being the algorithm was designed to only use the type and the duration. The augmentation was done for notes and structured entries of the same patient on the same day. Note entries that matched a structured entry in both type and duration were considered duplicates and excluded. Although such notes could represent additional entries, we prioritized reducing false positives by omitting notes with identical information, even at the cost of potentially increasing false negatives. A timestamp-based comparison was also considered unreliable, as patients may record notes and structured entries at varying times throughout the day. In our dataset we did not identify the case that duration and type were identical, but the intensity differed between structured entries and notes.

If neither parameter was identical with a structured entry on the same day, the note was interpreted as a missing entry. If one parameter matched, the note was considered a correction.

First, note entries that included time shifts (e.g., “yesterday”) were used to correct the date of structured entries with the same activity type.

We then iterated over each note entry individually to compare one note entry to all available structured entries from that day at a time.

- If no structured entry was available a new entry was created.
- If both type and duration matched, the note was considered a duplicate.
- If only the type matched, the duration corrected the structured entry.
- If the type did not match, the note was added as a new entry.

Structured entries which have already been corrected once, were no longer editable, so that e.g., two note entries with the same type did not alter the duration of one structured entry twice but correct once and add a new activity for the second note entry.

If a note explicitly indicated that a structured entry was incorrect or not actually performed, that structured entry was deleted.

The rule-based augmentation of existing data was carried out in Matlab.

### 2.5 LLM pipeline

The notes contained sensitive information ranging from medication and the state of health to personal information like names and contact information. Hence, despite prior pseudonymization, a local instance of an LLM was required. We applied a local instance of the Mistral-7B-Instruct-v0.3 model with 7.25B parameters (26). This model was deployed in the AI infrastructure of the AIT and accessible through a python-based toolkit developed by the AIT. For a comparison with state of the art models, the ChatGPT model gpt-4.1-2025-04-14 with 1.8 trillion parameters was used for benchmarking on the synthetic notes. For consistency and fairness, the GPT-4.1 analysis used the best performing prompts for each category of the Mistral model without modification. Structured data augmentation was not evaluated with synthetic data, as we could not ensure that the local LLM adequately captured the complex relationships between clinical notes and structured entries to produce a valid synthetic dataset. The AIT developed a toolkit to evaluate and run experiments with large language models running locally or remotely, which was used during this work.

### 2.6 Prompt engineering

We followed the idea of few-shot prompting as described in (27). A separate prompt was designed for each of the parameters, the overall classification (activity-related yes/no), and the augmentation of the structured PA data. Within each prompt, the required outcome formats were provided. To streamline the evaluation of the LLM responses, LLM results were demanded as JSON objects with a key value pair that stores the extracted information in an array. Additionally, the LLM was specifically instructed not to provide explanations for its decision, since only the structured key value pairs were considered by the subsequent evaluation algorithm. Multiple prompts were written for each of the parameters and tasks, including different phrasings and examples, all following the concept of few-shot prompting. This paper only presents the best performing prompt for each task. The activity types extracted by the LLM have been checked manually before the evaluation, since the model sometimes returned English types even though the notes and hence the gold standard was in German. Additionally, as for the regex algorithm, slight variations of essentially the same exercise [e.g., “gehen” (walking) and “spazieren” (leisurely walking)] were combined. For the extraction of durations, the LLM was instructed to only retrieve durations which were either directly related to activity types or to other PA measures, such as distance or heart rate. The LLM was also instructed to convert the extracted durations to minutes, to ensure comparability with the gold standard. The prompt to extract the intensity was designed to only extract PA related intensities. Like the regex algorithm, the prompt for the separation of activity from non-activity-related notes was formulated very generally, giving the LLM the freedom to interpret the notes without too many restrictions.

The LLM was given a prompt with a task definition, the note and the structured PA data for task 2. Just like the rule-based algorithm, the LLM was given all notes and all structured PA entries of a single patient on the same day as input.

### 2.7 Test set annotation

To evaluate the performance of the algorithms, a subset of 1,000 randomly selected notes from 112 patients was annotated manually. These notes were withheld from the development of the rule-based algorithm and, additionally, no other notes from these 112 patients were used for the development of the regex to avoid train test leaks. These 1,000 notes were annotated in a first round by three independent digital health specialists with domain knowledge, and in a second round, another expert decided on all notes where the annotators of the first round had given differing annotations to define our gold standard. For the data augmentation task an inter-annotator reliability was calculated using the test set annotations. For the metrics of the inter-annotator reliability, the first-round annotations were compared with themselves and whenever there was a disagreement, the final gold standard was used to determine whether the entry was a false positive or negative.

### 2.8 Statistical analysis

The evaluation of the algorithms’ performance in all three tasks was carried out in two steps:

- Detection of parameters: In this step, we only checked whether the algorithm detected any information in a note, without comparing the actual content (e.g., whether the algorithm detected duration information whenever a duration was annotated in the golden standard). This reduced the task to a simple yes/no classification.
- Correctness of the extracted parameters: In this step, we evaluated whether the content of the extracted information corresponded to the content of the annotations (e.g., whether the extracted duration of the algorithm corresponded to the duration in the golden standard). This was done only for notes where both the algorithm and human annotators had extracted the respective parameters.

The extraction of numerical values was framed as a classification task (correct value extracted or not), rather than by calculating the error between extracted and annotated durations, since our focus was on whether the algorithm could identify the exact number, not merely approximate it. Confidence intervals were calculated using bootstrapping with 1000 iterations. Normal distribution of bootstrapped F1-scores was tested with the Shapiro–Wilk test and statistical significance was tested with the following tests:

- Two groups
  - Normal distribution: T-Test (T)
  - Non-normal distribution: Mann–Whitney-U-Test (U)
- Three groups
  - Normal distribution: One-way ANOVA (A)
  - Non-normal distribution: Kruskal–Wallis-Test (K)

### 2.9 Ethics

This work was approved by the ethics committee of the Medical University of Vienna (vote number 1289/2024).

## 3 Results

### 3.1 Key statistics of the NLP analysis

**Figure 2:**
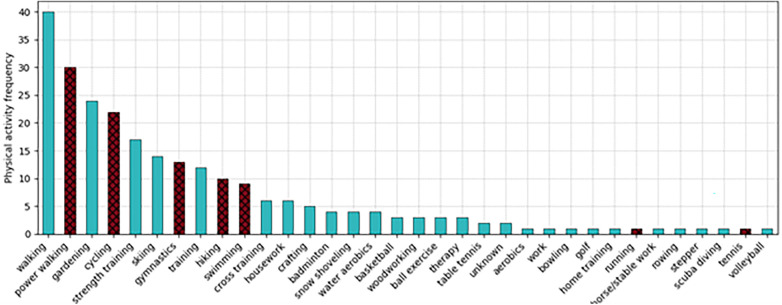
Distribution of the 34 manually annotated activity types in the validation set. Selectable activities in the DiabMemory app ‘are highlighted in red and with a crosshatch pattern.

Out of 1000 annotated free-text notes, 377 were manually classified as activity related. Of these, 230 comments had an activity “type”. Additionally, 12 free-text notes contained multiple types, resulting in a total of 248 extracted activity types across 34 different activities groups. The two activity notes annotated with the type *unknown* both mentioned that a vital parameter was measured after physical exertion without further specification. The distribution of these types is shown in Figure 2. In this figure, the seven activity types highlighted in red with a crosshatch pattern can also be uploaded as structured activity data (i.e., the seven types provided by the DiabMemory app). Overall, only three instances of intensity were annotated in the notes. Furthermore, 49 notes were manually identified as containing a duration for a physical activity, with five of these notes containing two durations each, for a total of 54 individual durations.

### 3.2 Task 1 – information retrieval and classification

#### 3.2.1 Task 1.1 – activity classification

 Table 1 presents the results of the activity classification described in task 1.1. For this task only the first step of the statistical analysis was necessary (i.e., Detection of parameters in Section 2.8), since no information was extracted during this stage.

**Table 1:** Results of the regular expression-based algorithm (regex) and mistral model for the activity classification (task 1.1).

Algorithm	TP	TN	FP	FN	Precision	Recall	Spec	Accuracy	F1-score
Activity classification									
Regex	363	617	16	4	98.91%	95.78%	99.36%	98.00%	.97 (95% CI:.96–.98) *p* < 0.001; U
Mistral	346	582	39	33	89.87%	91.29%	93.72%	92.80%	.90 (95% CI:.88–.93)

True Positives (TP), True Negatives (TN), False Positives (FP), False Negatives (FN), Specificity (Spec), p-value (p), Mann–Whitney-U-Test (U).

#### 3.2.2 Task 1.2 – primary parameters

 Table 2 presents the evaluation of the extraction of the primary parameters using the regex algorithm and the Mistral model as described in task 1.2. As can be seen in the Supplementary appendix under *A4 Duration prompt,* the prompt for the extraction of the duration was the only German prompt since in all other cases, English prompts yielded better results.

**Table 2:** Results of the regular expression-based algorithm (regex) and mistral model for the extraction of the primary parameters (task 1.2).

	Detection of parameters									Correctness	
Algorithm	TP	TN	FP	FN	Precision	Recall	Spec	Accuracy	F1-score	n correct	% correct
Type											
Regex	212	768	2	18	99.1%	92.2%	99.7%	98.0%	.96 (95% CI:.93–.97) *p* < 0.001 U	202	95.3%
Mistral	175	701	70	54	71.4%	76.4%	90.9%	87.6%	.74 (95% CI:.70–.78)	148	84.6%
Duration											
Regex	47	945	6	2	88.7%	95.9%	99.4%	99.2%	.92 (95% CI:.86–.97) *p* < .001; U	47	100%
Mistral	44	914	37	5	54.3%	89.8%	96.1%	95.8%	.67 (95% CI:.58–.77)	35	79.6%
Intensity											
Regex	1	997	0	2	100%	33.3%	100%	99.8%	.50 (95% CI: 0-1) *p* < 0.01; U	1	100%
Mistral	1	987	10	2	9.1%	33.3%	99.0%	98.8%	.14 (95% CI:0–.40)	1	100%

True Positives (TP), True Negatives (TN), False Positives (FP), False Negatives (FN), Specificity (Spec), p-value (p), Mann–Whitney-U-Test (U).

#### 3.2.3 Task 1.3 – secondary parameters

 Table 3 presents the evaluation of the extraction of the secondary parameters using the regex algorithm and the Mistral model as described in task 1.3.

**Table 3:** Results for the regular expression-based algorithm (regex) and mistral model for the extraction of the secondary parameters (task 1.3).

	Detection of parameters									Correctness	
Algo	TP	TN	FP	FN	Precision	Recall	Spec	Accuracy	F1 Score	n correct	% correct
Distance											
Regex	98	899	2	1	98.0%	99.0%	99.8%	99.7%	.99 (95% CI:.96–1) *p* < .001; U	96	98.0%
Mistral	91	816	84	9	52.0%	91.0%	90.7%	90.7%	.66 (95% CI:.60–.73)	63	69.2%
Elevation change											
Regex	8	992	0	0	100%	100%	100%	100%	1 (95% CI: 1-1) *p* < .001 T	8	100%
Mistral	8	962	30	0	21.1%	100%	90.2%	97.0%	.35 (95% CI:.16–.50)	8	100%
Heart Rate											
Regex	105	895	0	0	100%	100%	100%	100%	1 (95% CI: 1-1) *p* < .001; U	105	100%
Mistral	105	880	15	0	87.5%	100%	98.3%	98.5%	.93 (95% CI:.90–.96)	104	99.1%
Calories											
Regex	106	892	1	1	99.1%	99.1%	99.9%	99.6%	.99 (95% CI:.98–1) *p* < .001;U	103	97.2%
Mistral	108	874	18	0	85.7%	100%	98.0%	98.2%	.92 (95% CI:.88–.96)	105	97.2%
Steps											
Regex	17	981	0	2	100%	89.5%	100%	99.8%	.94 (95% CI: 0.85-1) *p* < .001; U	17	100%
Mistral	19	974	7	0	73.1%	100%	99.3%	99.3%	.85 (95% CI:.72–.94)	19	100%
Watt											
Regex	4	996	0	0	100%	100%	100%	100%	1 (95% CI: 1-1) *p* < .001;U	3	75.0%
Mistral	3	978	18	1	14.3%	75.0%	98.2%	98.1%	.24 (95% CI:.08–.51)	3	100%

True Positives (TP), True Negatives (TN), False Positives (FP), False Negatives (FN), Specificity (Spec), p-value (p), Mann–Whitney-U-Test (U).

### 3.3 Task 2 – augmentation of existing data

Table 4 shows the results of the rule-based augmentation algorithm, and the Mistral model-based augmentation compared to the gold standard. Table 4 also shows the inter-annotator reliability to demonstrate the complexity of the augmentation task.

**Table 4:** Results for the rule-based algorithm and mistral model for the extraction of the secondary parameters (task 1.3).

	Detection of parameters									Correctness	
Algorithm	TP	TN	FP	FN	Precision	Recall	Spec	Accuracy	F1 Score	n correct	% correct
Rule-based	201	964	79	67	71.8%	75.0%	92.4%	88.9%	.73 (95% CI:.69–.77) *p* < .001; K	173	86.1%
Mistral	175	895	494	94	26.2%	65.1%	64.4%	64.5%	.37 (95% CI:.33–.41)	79	45.1%
Reliability	102	957	55	136	65.0%	42.9%	94.6%	84.7%	.52 (95% CI:.46–.58)	89	87.3%

Additionally, the inter-annotator reliability is shown. True Positives (TP), True Negatives (TN), False Positives (FP), False Negatives (FN), Specificity (Spec), p-value (p), Mann–Whitney-U-Test (U).

### 3.4 Task 3 – information retrieval and classification with synthetic data

#### 3.4.1 Task 3.1 – activity classification

 Table 5 presents the results of the activity classification of the synthetic data described in task 3.1 with the addition of the GPT 4.1 model.

**Table 5:** Results of the regular expression-based algorithm (regex), mistral and GPT model for the activity classification (task 3.1).

Algorithm	TP	TN	FP	FN	Precision	Recall	Spec	Accuracy	F1 Score
Classification									
Regex	67	31	0	2	100%	97.1%	100%	98.0%	.99 (95% CI:.92-1) *p* < .001; K
Mistral	29	45	22	4	56.9%	87.9%	67.2%	73.5%	.69 (95% CI:.56-.78)
GPT	32	38	29	1	52.5%	97.0%	56.7%	70.0%	.68 (95% CI:.57-.79)

True Positives (TP), True Negatives (TN), False Positives (FP), False Negatives (FN), Specificity (Spec), p-value (p), Mann–Whitney-U-Test (U).

#### 3.4.2 Task 3.2 – primary parameters

 Table 6 presents the evaluation of the extraction of the primary parameters using the regex algorithm, the Mistral and the GPT model as described in task 3.2.

**Table 6:** Results of the regular expression-based algorithm (regex), mistral model and the GPT model for the extraction of the primary parameters on the synthetic data set (task 3.2).

	Detection of parameters									Correctness	
Algorithm	TP	TN	FP	FN	Precision	Recall	Spec	Accuracy	F1 Score	n correct	% correct
Type											
Regex	22	76	0	2	100%	91.7%	100%	98.0%	.96 (95% CI:.89–1) p < .001;K	22	100%
Mistral	20	76	0	4	100%	83.3%	100%	96.0%	.91 (95% CI:.81–0.98)	20	100%
GPT	18	76	0	6	100%	75.0%	100%	94.0%	.86 (95% CI:.73–.96)	18	100%
Duration											
Regex	5	95	0	0	100%	100%	100%	100%	1 (95% CI: 1-1) p < .001; K	5	100%
Mistral	5	84	11	0	31.3%	100%	88.4%	89.0%	.48 (95% CI:.14–0.67)	5	100%
GPT	5	95	0	0	100%	100%	100%	100%	1 (95% CI: 1-1)	5	100%
Intensity											
Regex	0	98	0	2	N/A	0.0%	100%	98.0%	N/A p = N/A	0	N/A
Mistral	2	97	1	0	66.7%	100%	99.0%	99.0%	.80 (95% CI: 0–1)	2	100%
GPT	1	98	0	1	100%	50.0%	100%	99.0%	.67 (95% CI: 0–1)	1	100%

True Positives (TP), True Negatives (TN), False Positives (FP), False Negatives (FN), Specificity (Spec), p-value (p), Mann–Whitney-U-Test (U).

### 3.5 Runtime difference between approaches

The runtime of both models was tested on a test run of 10 iterations to evaluate the time needed to compute the test set of 1,000 notes to show practical implications for a real world deployment. During this test run the regex algorithm needed 22.29 (±1.65) seconds for the retrieval of all primary and secondary parameters and the classification of 100 notes, run on a 12^th^ Gen Intel(R) Core (TM) i5-1245U.

The locally deployed Mistral LLM model within the AIT LLM pipeline required 309.78 (±159.69) seconds to classify and retrieve information for a single task when executed on the AIT infrastructure. Extrapolated to all 10 tasks, this corresponds to a total runtime of 3,097.80 seconds. In comparison, the regex-based algorithm would complete the same analyses in only 0.72% of the time required by the AIT LLM pipeline.

## 4 Discussion

This paper evaluates the performance of two privacy-preserving, locally deployed NLP algorithms for extracting physical activity–related information from real-world free-text notes of diabetes patients. In addition, these algorithms were compared to a cloud-based state-of-the-art LLM using synthetic data. The analysis provides deeper insights into the dataset and underscores the potential of applying NLP to patient notes. The variety of activity types reported by DiabMemory patients highlights the potential of NLP analysis and offers valuable insights for future updates of the structured PA entry system. The distribution of activities also reflects the demographics of the DiabMemory cohort, with walking, cycling, and gardening being among the most common exercises, while the frequency of skiing reports reflects the Austrian context of the system. Manual annotation further revealed that not all parameters are equally represented in the notes, since many entries included information on activity type, duration, heart rate, calories burned, and distance, whereas intensity and wattage were rarely reported. The activities most frequently reported in the free-text notes fell outside the predefined list of activity types in the DiabMemory app. This is unsurprising, as patients are expected to use the structured options whenever possible, resorting to free-text entries only when no suitable structured type is available.

Performances varied across tasks and algorithms but generally showed promising results, with numerical values being easier to classify and extract than the categorical variable “type”. According to our results, the Mistral LLM consistently produced more false positives than the regex algorithm across all analyses. It also tended to infer information from context, even though the prompt explicitly instructed it to extract only information explicitly stated in the notes. This issue was particularly evident for the ‘type’ and ‘distance’ categories, which showed substantially higher false-positive rates compared to the regex approach. For distance, in particular, the LLM often failed to distinguish between true distance values and other numerical information, such as steps, calories, or unrelated values like vital parameters. This issue persisted even when explicitly instructing the LLM that numerical values combined with terms such as “Schritte” (steps) or “Kcal” (kilocalories) should not be interpreted as distance measurements. The Mistral model frequently inferred activity types from notes containing exercise-related numbers, even when no explicit activity type was provided. For example, it hallucinated „cycling“ from a note simply reading „3, 7km–50min“ or „driving“ from „fahre auf kurzurlaub“ („going on a short trip“), neither of which represents a physical activity or an explicit physical activity type. This behavior occurred despite instructions to extract activity types only when they were explicitly stated and clearly related to physical activity.

Regex-based algorithms produced fewer false positives, though this approach has limited capacity for contextual interpretation. When comparing correctly extracted information across all true positive notes, the regex algorithm either matched or exceeded the accuracy of the Mistral model. Categories such as intensity, elevation change, and watt had very few positive cases, limiting the generalizability and statistical power of these results. The extraction of exercise distances revealed a discrepancy between the algorithms, with the regex approach achieving 100% accuracy compared to 69.23% for the Mistral model.

The Mistral model showed difficulties in distinguishing between distances and other exercise related numerical parameters. The note “4, 8 km, 318 hm” resulted in [4.8, 318], both as extracted distance parameters. Additionally, the LLM showed inconsistencies in handling comma separated distances meaning that “6, 1 Kilometer” was extracted as “6”, “2, 11 Km; 200 Kcal; Puls 80 - 100;” was extracted as “11” and “6, 73 Km; 342 Kcal; Puls 95–108” was extracted as “673”. Apparently, the Mistral model did not interpret the variations of commas and spaces correctly, even though this issue was addressed during prompt engineering and examples with possible notations were included. Since the examples given above followed the same notation but resulted in different results, a systematic error during prompt engineering does not seem to be the issue. The secondary PA parameters exhibited consistent notation in the DiabMemory notes; for example, heart rate during exercise was almost exclusively recorded as e.g., 100-120 Puls (i.e., “Number – Number Puls”). These recurring patterns enabled the regex algorithm to perform very well. However, this also implies that the performance of the regex algorithm on other datasets with less uniform notation should be evaluated in future work, as it may worsen with more variety in the notation.

Although the regex algorithm outperformed the local Mistral LLM in both accuracy and computational efficiency, its development remains complex and time-consuming, requiring prior in-depth knowledge of the dataset. In contrast, deploying the pre-trained Mistral model did not require such dataset-specific development. Even though multiple prompts per task were developed and evaluated, the time spent on prompt engineering was substantially lower than the effort needed to create the regex rules. Additionally, unlike the regex approach, the Mistral LLM required more manual post-processing to ensure machine-readable outputs, as it often produced JSON-like results with invalid formatting.

To ensure a fair comparison between the two algorithms, the activity types have been manually checked to ensure coherence with the gold standard, meaning that Mistral LLM outputs like *radfahren (cycling)*, *ergometer* or *mountainbiking* all resulted in true values for the gold standard *radfahren (cycling)*. Additionally, even though the Mistral LLM was instructed to refrain from any logical explanation and information apart from the structured key value pairs, the model tended to occasionally add such information regardless, which had to be considered in the post processing.

Even though the regex algorithm outperformed the Mistral LLM in the augmentation task, the results of this augmentation task illustrated the complexity of the underlying issue. The relatively moderate inter-annotator reliability with an F1-score of.52 emphasized this even further. While initially appearing straightforward, the task of mapping PA information extracted from unstructured free-text notes to structured PA data rapidly increased in complexity due to the multiple degrees of freedom and ambiguity of the notes. Especially distinguishing whether a note refers to an existing structured entry or a new entry which has not yet been reported leads to many discrepancies in the annotation. However, the results also show that in cases where all annotators changed a structured entry the agreement between the changes was high with an accuracy of 87.25%. Additionally, most of the notes were missing a broader context, which might have been more obvious if the entire patient – physician communication had been known. The regex algorithm was designed to only cover obvious cases for augmentation, e.g., one to one matching between a note and a structured entry with a missing parameter corresponding to the extracted parameter from the note. Highly complex cases were intentionally omitted by the regex algorithm due to its lack of understanding of an underlying context or general ambiguity of the notes. We assume that this is the reason why the rule-based algorithm was able to achieve a higher F1-Score than the annotators for the inter-annotator reliability. Applying the rule-based algorithm to a different dataset might show signs of a potential overfitting. Given the fact that the broader context is missing in this setting, an LLM might perform better than a regex-based algorithm in other settings with longer input notes and more context.

Our corpus contained only German free-text notes. Therefore, for application on corpuses in other languages, the regex patterns need to be translated and adapted. Some of the prompts would likewise need to be translated into English.

To compare the local algorithms with state-of-the-art LLMs a synthetic dataset, generated with the Mistral LLM was used. The GPT 4.1 model was outperformed by the regex algorithm in all categories except intensity. However, the GPT model was able to significantly outperform the local Mistral LLM in the extraction of durations only (*p* < .005; U). The prompts used for both LLMs were optimized for the Mistral model, and the synthetic texts were also generated by Mistral, which leads to some bias in favor of Mistral over GPT in the evaluation. A closer look at the extracted activity types revealed that 5 of the 6 types of false negatives of the GPT model were part of the categories *hausarbeit (housework)* and *gartenarbeit (gardening)*. Whilst the Mistral model did recognize those activities without the need for an explicit statement in the examples of the prompt, the GPT model did not include these types without an explicit mention in the examples. This indicates that with more extensive prompt engineering, the GPT model might outperform the Mistral LLM and potentially even the regex algorithm. However, the focus of this work was not to optimize the prompts for a state-of-the-art LLM but explore and optimize local algorithms, hence for this comparison the limited prompt engineering for the GPT model must be taken into consideration. Additionally, the synthetic dataset used for comparison with the state-of-the-art GPT model was limited to 100 notes, a very small sample size. Since the notes were generated by the local Mistral LLM, Mistral may have held an inherent advantage in processing data it had created itself. However, this bias is not likely since the Mistral model did not perform better than the other tested algorithms. Additionally, during the annotation process the experts validated the generated notes to be similar to the real-world data and removed notes with wrong or uncommon phrases and structures. Nevertheless, circular bias cannot fully be ruled out and, therefore, the results of the synthetic data analysis must be handles with care.

The regex algorithm showed significantly lower processing times than the LLM pipeline used for these analyses which is an important factor when thinking about real time analysis of the data in a live service. However, runtime optimization was not a priority during algorithm and LLM pipeline development.

Wiest et al. evaluated local LLMs ranging from 7B to 70B parameters for information extraction from medical free text using zero- and one-shot prompting, a strategy also applied in this work. They reported that the smallest model (7B parameters, comparable in size to the Mistral model used during this work) performed worse than larger models, supporting our assumption that a larger model would likely yield better results in analyzing the DiabMemory notes. Although the local Mistral LLM used in this work has a relatively small size of 7.25B parameters compared to most state-of-the-art models, computational resources remain limited in some healthcare settings as well as in the infrastructure available for this study

Kaster et al. and Patra et al. found that rule-based algorithms outperformed LLMs across most evaluated parameters (22, 23). Our results are consistent with these findings, since in tasks 1 and 2, rule-based approaches outperformed their LLM counterparts. However, Kaster et al. also noted that rule-based methods exhibited weaker generalizability than LLMs (22). This limitation may also apply to our algorithms, which were specifically tailored to extract PA information from telehealth notes of diabetes patients. Further work is required to evaluate their generalizability and applicability in different patient cohorts and different telehealth settings.

Furthermore, consistent with Sivarajkumar et al., we observed that the Mistral LLM in our study frequently produced false positives, resulting in lower precision and F1-scores compared to the rule-based approach (24).

Although we annotated nearly twice as much free text as Wiest et al. and Patra et al., our test set remained small compared to Sivarajkumar et al., who analyzed 23,724 clinical notes (21, 23, 24). A larger sample size would have enabled a more powerful and robust evaluation of all parameters and especially of infrequent parameters such as intensity or workload. Nevertheless, the more relevant parameters like type and durations had plenty of positive cases in the test set and because the sparse parameters are expected to be equally sparse across the entire dataset, their limited impact on real-world data reduces the practical consequences of the small test set.

A limitation which has not been addressed in the previous work was the runtime of the algorithms. As shown in Section 3.5 the Mistral model was drastically slower in processing the notes, since every note was processed as a separate prompt. During routine implementation this could be a potential drawback, just like the availability of the LLM, especially if multiple users try to execute prompts simultaneously. In contrary, the regex algorithm could be run on local machines with significantly lower computational power, without concerns for server availability or response times.

## 5 Conclusion

This work shows the potential of NLP for extracting PA information from free-text notes in a diabetes telemedical system. Among the two locally deployed NLP methods evaluated, the rule-based algorithms showed better accuracy across all tasks and lower runtimes as compared to our local LLM. Although rule-based approaches require more background knowledge and longer development time, they remain a viable option for local NLP, capable of surpassing local LLMs in certain contexts without the high computational costs associated with large models.

## Data Availability

The data sets generated during and/or analyzed during this study are not available due to privacy concerns and based our ethics approval and the initial informed consent. Requests to access the datasets should be directed to Fabian Wiesmüller, fabian.wiesmueller@ait.ac.at.

## Footnotes

^1^ https://de.mathworks.com/products/matlab.html

^2^ https://de.mathworks.com/products/matlab.html
