# Supplementary material for: Privacy-preserving augmentation of structured telehealth activity data in diabetes patients using natural language processing
Source: Front Digit Health. 2026 May 7;8:1720149. doi: 10.3389/fdgth.2026.1720149 (PMC13190399; doi:10.3389/fdgth.2026.1720149)
Supplement: Supplementary file 1 [file Supplementaryfile1.pdf]

## A1 Regular expressions

<table>
<tr><th>Number</th><th>Regex</th></tr>
<tr><td>1</td><td>garten(\-)?arb\w*|gartenpflege|gartentätigkeit|gartenumgrab\w*|gartensrbeit|garten\sarbeit|im\sgarten\sgearbeitet|(rasen\s?)?(ge)?m(ä|ae)h(t|en)|(^|\s)(ge)?rech(en|t)(?!zeitig)|hecken?\s?(ge)?(schnitt(en)?|schneiden)|\w*ernte|weinlese|arbeit\w*\sim\s(haus|heim)garten</td></tr>
<tr><td>2</td><td>\w*(?<!tisch)tennis</td></tr>
<tr><td>3</td><td>tischtennis</td></tr>
<tr><td>4</td><td>(\w*ergo(mo)?met(er|rie)|\d{1,3}\smin.{1,3}rad|\w*rad(\s?|ge)fahren|zimmer\w*(fahr)?rad|(fahr)?rad\saus(gefahren|fahrt)|radtour\w*)|(ergo\w*(\-|\s?)training)|((home?|heim)\s?tr(ai|a|ae|ä)n\w*)|(fahre.{1,13}rad($|\s))|(^|\s)radf\.(\s|$)|(ergo\sfit)|belastungs\s?\-\s?ekg</td></tr>
<tr><td>5</td><td>(laufen|laufband|gelaufen|(\s|^)lief|\w*marathon|jogg(ing|en)|dauerlauf)</td></tr>
<tr><td>6</td><td>(ge)?walk(en|t|ing)|schnell(es)?\sgehen|nordi(c|k)(?!\s?walk\w*)</td></tr>
<tr><td>7</td><td>(((vor\w*|nach\w*|bin|war|heute|\w*gestern|\d\s?tagen|\w*morgen|uhr|stunden|std\.?|h).{1,6})?(wander\w*|gewandert)|bergtour\w*|^wandern$|(wald|berg|alm)\s?wanderung|\w*wandertag|berggehen)</td></tr>
<tr><td>8</td><td>\w*tanzen</td></tr>
<tr><td>9</td><td>tanz\w*</td></tr>
<tr><td>10</td><td>(\w*fitness\w*|fitness\s?(center|studio))</td></tr>
<tr><td>11</td><td>((ski|schi)(ge)?\s?\-?\s?((lang)?lauf(en)?|fahren|tour\w*)|ski\s?alpin|touren\s?ski)</td></tr>
<tr><td>12</td><td>volle?yball</td></tr>
<tr><td>13</td><td>(eisstock\w*|stockschie(ss|ß)\w*)</td></tr>
<tr><td>14</td><td>\w*bike\w*</td></tr>
<tr><td>15</td><td>tr.\s?therapie|(bewegungs|physio)\s?therapie|cor\s?ther(\.|$|\w*)</td></tr>
<tr><td>16</td><td>rodeln</td></tr>
<tr><td>17</td><td>(\w*(?<!aus)schwimm(?!brille)\w*|geschwommen|schwamm(\s|$)|\d+\sl(ae|ä)ngen|kraul(\.|\w*)|min\.?\sbrust|im\spool)</td></tr>
<tr><td>18</td><td>(\w*tauch(t|en))|(schnorcheln)|(tauchgang)</td></tr>
<tr><td>19</td><td>golf\w*</td></tr>
<tr><td>20</td><td>(\w*(?<!aqua|wasser|ski|schi)gymnasti(k|c)\w*|boden\s?gym\w*|(\s|^)gymn(\.|\s|$)|gymn(\.|\;|\d))</td></tr>
<tr><td>21</td><td>(wasser|aqua)\s?gym\w*</td></tr>
<tr><td>22</td><td>(ski|schi)gym\w*</td></tr>
<tr><td>23</td><td>\w*turnen\w*</td></tr>
<tr><td>24</td><td>bauarbeiten</td></tr>
<tr><td>25</td><td>(waldarbeit|baumstutzen|holz(ge)?hack(en|t))|b(au|äu|aeu)me?\s(ge)?f(ae|ä)ll(t|en)|\w*holz(auf|\s)?arbeit(en|ung)?</td></tr>
<tr><td>26</td><td>aerobik</td></tr>
<tr><td>27</td><td>(schnee\s?(ge)?schaufel(t|n))|(schnee\s?(ge)?r(ae|ä|a)um(en|t))</td></tr>
<tr><td>28</td><td>(kegel\w*)|(gekegelt)|(bowling)|(gebowled)|(kegelabend)</td></tr>
</table>

<table>
<tr><td>29</td><td>rafting</td></tr>
<tr><td>30</td><td>(haus\s?(arbeit\w*|putz\w*)|arbeit\w*(\sin)?(\sder)?\s?wohnung|whonungs\s?arbeit|putzen|auf(ge)?r(ä|ae|a)um(en|t)|arbeit\w*\s(im|am)\shaus)</td></tr>
<tr><td>31</td><td>(ge)?mal(en|erei|t)</td></tr>
<tr><td>32</td><td>(^|langsam\w*\s)gehen$|(km|min|schritte)\sgegangen|schritte\sunterwegs|gassi\s?gehen|(\w*spa?z?ie?r(g|q)an(q|g)|\d*\s?\.?\s?min\sgehen|(?<!steil\s)bergauf(?!\swalk\w*)|fu(ss|ß|s)marsch|(schwammerl|pilze)\s?(ge)?(such|sammel)(en|t))</td></tr>
<tr><td>33</td><td>((spazier(en|t)(?!.*mit\shund)|mit\s?(dem\s)?hund)(?!.*mit\s(dem\s)?hund)|gehe\st(ae|a|ä)glich\smit\sdem\shund|besoe?rgungen)</td></tr>
<tr><td>34</td><td>(kraft(k|t)\w*|kraftraining|maschine\w*|maschine.*(brust|beine|bauch)|ger(ä|ae)te|muskel\s?training|(^|\s)kraft($|\s|,|;)|r(u|ue|ü)cken\s?training)</td></tr>
<tr><td>35</td><td>(badminton|badmington|federball)</td></tr>
<tr><td>36</td><td>(morgensport|(bei|nach)(m)?\ssport|sport\sbe)</td></tr>
<tr><td>37</td><td>yoga</td></tr>
<tr><td>38</td><td>(crossger(ae|ä|a)t|crosstr(ae|ä|ai|a)n\w*|spinning)</td></tr>
<tr><td>39</td><td>handergometer</td></tr>
<tr><td>40</td><td>basketball\w*</td></tr>
<tr><td>41</td><td>ausdauer\w*</td></tr>
<tr><td>42</td><td>(aufwär\w*|aufwaerm\w*|dehnen)</td></tr>
<tr><td>43</td><td>#####\d{1,3}\smin\s\[per-.{8}\]#####DoNotUse#####</td></tr>
<tr><td>44</td><td>stair\w*</td></tr>
<tr><td>45</td><td>ausk(ü|ue)hlen</td></tr>
<tr><td>46</td><td>cool(\-|\s)?down</td></tr>
<tr><td>47</td><td>(step{1,2}er|\d{1,3}\smin\sstepp?)</td></tr>
<tr><td>48</td><td>(?<!\[per\-.{8}\]\s)\w*-?train(ing\w*|er|ier)\w*</td></tr>
<tr><td>49</td><td>\[per\-.{8}\]\strain\w*</td></tr>
<tr><td>50</td><td>(?<!schmerzen\s)(nach\w*\s)?belastung</td></tr>
<tr><td>51</td><td>werken|gewerkt|(ge)?(b|p)astel(n|t)</td></tr>
<tr><td>52</td><td>rudern</td></tr>
<tr><td>53</td><td>sitzball</td></tr>
<tr><td>54</td><td>fu(ss|ß)ball\s?(training|(ge)?spiel(t|en))</td></tr>
</table>
