# Supplementary material for: Privacy-preserving augmentation of structured telehealth activity data in diabetes patients using natural language processing
Source: Front Digit Health. 2026 May 7;8:1720149. doi: 10.3389/fdgth.2026.1720149 (PMC13190399; doi:10.3389/fdgth.2026.1720149)
Supplement: Supplementary file 2 [file Supplementaryfile2.pdf]

A2 Classification Prompt

Kontext: You are a tool that flags german free text elements as activity related (1) or not (0). No additional output, just a key value pair like "activity_related":result
Free text: {prompt}

A3 Type prompt

Instruction:

You are a tool that extracts explicitly stated types of physical activity or exercise from German free-text input. Follow these rules:

Extract only explicitly mentioned activity or exercise types (e.g., Joggen, Radfahren, Schwimmen).

Do not infer the type based on related metrics like duration, distance, elevation, or calories unless a type is explicitly named.

Multiple types may be present. Return all in a list.

If no activity type is mentioned, return none.

Respond only with a JSON-style key-value pair:

"activity_type": [result]

Where [result] is a list of activity types (as strings), or none.

Examples:

"Heute 5 km Joggen, danach 30 Minuten Radfahren." → "activity_type": ["Joggen", "Radfahren"]

"Ich war schwimmen und habe danach noch Yoga gemacht." → "activity_type": ["Schwimmen", "Yoga"]
"Bin heute 10 km gelaufen." → "activity_type": ["Laufen"]
"700 Kalorien verbrannt, 400 Höhenmeter überwunden." → "activity_type": none
"Wanderung abgesagt wegen Regen." → "activity_type": none

Free text: {prompt}

A4 Duration prompt

Kontext: Du bist ein Werkzeug, das Zeitangaben (in Minuten) zu körperlichen Aktivitäten oder Übungen aus deutschen Freitexten extrahiert.

Regeln:
Extrahiere nur Zeitangaben die direkt mit körperlicher Aktivität oder Sport zusammenhängen
Extrahiere alle Zeitangaben in Minuten (z.B. „30 Minuten“, „eine halbe Stunde“, „1h“).
Gib alle gefundenen Werte in einer Liste zurück.
Falls keine Zeitangabe vorhanden ist, gib None zurück.
Gib nur ein JSON-ähnliches Ergebnis im Format "duration": result zurück (kein weiterer Text).

Beispiele:

Eingabe: „Ich war 30 Minuten joggen.“ → Ausgabe: "duration": [30]
Eingabe: „Ich habe eine halbe Stunde Yoga gemacht.“ → Ausgabe: "duration": [30]
Eingabe: „Putzen.“ → Ausgabe: "duration": None
Eingabe: „Strecke: 51, 5 km“ --> Ausgabe: "duration":None

Eingabe: „2, 3 km, 150 hm“ --> Ausgabe: "duration":None

Freitext: {prompt}

A5 Intensity prompt

Instruction: Extract the explicitly stated intensity level of physical activity from the following German free text. Valid intensity levels are: "low", "medium", "high".
Only assign an intensity if it is either explicitly mentioned (e.g., "hohe Intensität", "moderates Training").
If neither condition is met, return: "intensity": none.
Measurements of the heart rate should **not** be considered as intensity measures.
Do not infer intensity from vague phrasing or general context.
Return only a JSON-style key-value pair in the format: "intensity": [valid intensity level], or "intensity": [none].

Free text: {prompt}

A6 Distance prompt

Instruction: Extract the distance covered during exercise from German free texts.
This is usually indicated with a number (including **.** and **,** as separator) and the unit **km**, **kilometer**, **m** or **meter**. Multiple values are possible but uncommon. All distance values should be stored in the result as integers in a list.
If no distance value is in the text, the result should be none.
Example: "500 Hm; 2, 34 km; 130 Watt" --> [2.34]
Example: "Radfahren 15,2 kilometer" --> [15.2]
Example: "12.5 km gehen" --> [12.5]
Example: "laufen 2000 m" --> [2000]

No additional output/information, just a key-value pair like "dist": result

Free text: {prompt}

A7 Elevation change

Instruction: Extract elevation changes or elevation indications related to physical activities/exercises from German free texts. Common key indicators are e.g. **HM**, **Höhenmeter**, etc. Multiple values are possible. All elevation values should be stored in the result as integers in a list. If no elevation value is in the text, the result should be none.

Example: "Radfahren; 300 Höhenmeter! --> [300]

Example: "500 Hm; 250 kcal; Puls 130-155" --> [500]

Example: "Puls in Ruhe: 120" --> []

Example: "Wandern insgesamt 600 hm" --> [600]

Important: Numbers in combination with "Puls", "KM" and "Kcal" are NO elevation changes

No additional output/information, just a key-value pair like "elevation": result

Free text: {prompt}

A8 Heart Rate

Instruction: You are a tool that extracts heart rate values related to physical activities/exercises from German free texts. Common key indicators are e.g. Herzfrequenz, Puls, bpm, Schläge pro Minute, etc. Multiple values are possible. All heart rate values should be stored in the result as integers in a list. If the heart rate is specified as a range indicated by a *-* the range should be extracted. If no heart rate value is in the text, the result should be none.

Example: "Laufen; Puls be 150! --> [150]

Example: "500 Hm; 250 kcal; Puls 130-155" --> [130-150]

Example: "Puls in Ruhe: 120" --> []

Example: "Herzfrequenz bei training zwischen 100 und 120" --> [100-120]

Important: Numbers in combination with "KM", "HM", "Watt" and "Schritte" are NO heart rate values.

No additional output/information, just a key-value pair like "heart_rate": result

Free text: {prompt}

A9 Calories Prompt

Instruction: Extract the calories burned during exercise from German free texts. This is usually indicated with a number and the unit **kcal** or **kalorien**. Multiple values are possible but uncommon. All calorie values should be stored in the result as integers in a list. If no calorie value is in the text, the result should be none.
Example: "500 Hm; 250 kcal; 130 Watt" --> [250]
Example: "Ergometer 150kcal" --> [150]
Example: "120 Kalorien verbrannt bei Training" --> [120]
Example: "300 Kalorien; 500 Hm; 5 Km" --> [300]
Important: Numbers in combination with "Puls", "KM", "HM", "Watt" and "Schritte" are NO calorie values.
No additional output/information, just a key-value pair like "calories": result
Free text: {prompt}

A10 Steps

Instruction: Extract the nuber of steps taken from German free texts. This is usually indicated with a number and then followed by **Schritte**. Multiple values are possible but very uncommon. All steps values should be stored in the result as integers in a list. If no steps value is in the text, the result should be none.

Example: "500 Hm; 5000 Schritte; 130 Watt" --> [5000]

Example: "Heute 5000 schritte gegangen" --> [5000]

Example: "Gestern beim Laufen 2500 schritte gemacht" --> [2500]

Important: Numbers in combination with "Puls", "KM", "HM", "Watt" and "Kcal" are NO step values.

No additional output/information, just a key-value pair like "steps": result

Free text: {prompt}

A11 Watt

Instruction: Extract workload values during exercise from German free texts. This is usually indicated with a number and the unit **Watt**. Multiple values are possible but uncommon. All workload values should be stored in the result as integers in a list. If no workload value is in the text, the result should be none.

Example: "500 Hm; 250 kcal; 130 Watt" --> [130]

Example: "Ergometer mit 150 Watt" --> [150]

Example: "120 Watt bei Training" --> [120]

No additional output/information, just a key-value pair like "workload": result

Free text: {prompt}

A12 Augmentation

You are a tool that extracts information about physical activities or exercises from German free-text entries. In addition to these texts, you receive (semi-)structured activity entries with the non-mandatory parameters: type, intensity, and duration. Both the free texts and structured data are from the same person and the same day.

Task:

Your task is to process the inputs (free text and structured data) and determine whether:

An existing structured activity should be modified or completed (if values are missing),

A new activity should be added (even if incomplete),

Or an activity should be deleted (e.g. if it was entered incorrectly).

You must output a finalized list of activity entries, each containing the three

fields: type, intensity, and duration.

Important Instructions:

Do not infer missing data from context or phrasing.

All fields are mandatory in the output. If no information is available, leave the value blank (e.g., "").

Do not explain your reasoning.

Return only the final list as a JSON array of activity objects.

Free text(s): {prompt}. Structured data: {prd}
